# The Acari Hypothesis, IV: revisiting the role of hygiene in allergy

**DOI:** 10.3389/falgy.2024.1415124

**Published:** 2024-07-10

**Authors:** Andrew C. Retzinger, Gregory S. Retzinger

**Affiliations:** ^1^ Department of Emergency Medicine, Camden Clark Medical Center, West Virginia University, Parkersburg, WV, United States; ^2^ Department of Pathology, Feinberg School of Medicine, Northwestern University, Chicago, IL, United States

**Keywords:** the acari hypothesis, allergy, mites and ticks, α-gal hypersensitivity, human evolution, eccrine glands, sweat, hygiene

## Abstract

Allergy and its manifestations were first appreciated in the 1870 s. Today, the mechanism by which specific substances elicit allergic reactions remains poorly understood. This is problematic from a healthcare perspective because the prevalence of allergic disease and its societal costs are substantial. Regarding mechanistic understanding of allergy, a new proposal, The Acari Hypothesis, has been forwarded. The Hypothesis, borne from consideration of alpha-gal syndrome, postulates that acarians, i.e., mites and ticks, are operative agents of allergy. By way of their pathogenic payloads and salivary pattern recognition receptor(s), acarians potentiate in human hosts the generation of IgE against acarian dietary elements. Those elements account for most, if not all, known human allergens. Inasmuch as acarian—human interactions occur on human epithelial surfaces, it is to be expected factors that influence the presence and/or operation of acarians on those surfaces influence the expression of allergic diseases. In this report, it is proposed that two adaptations of catarrhine primates, i.e., Old World monkeys, apes and humans, evolved to deter acarian species: firstly, the expansion of eccrine glands across the entirety of body surface area, and, secondly, the secretion of sweat by those glands. Contemporary hygienic practices that reduce and/or disrupt the operation of eccrine glands are likely responsible for the increase in allergic disease seen today.

## 1 Introduction

“The Acari Hypothesis” is a multi-installment treatise that accounts for the cardinal pathophysiologic and epidemiologic features of IgE-mediated allergic disease. It presupposes that mites and ticks are the causative agents of allergy. The first installment of The Hypothesis makes the case that most allergens are elements of acarian diets (1). The second installment provides how such dietary elements, when transmitted to a human, elicit IgE (2). The third installment relates the first two to atopic dermatitis, the prototypical allergic disease (3). This installment of The Hypothesis, the fourth, provides rationale for the ongoing allergy epidemic. Importantly, IgE-mediated diseases have increased precipitously since 1870 (4), especially within developed countries. To date, no persuasive rationale for this increase exists. In the context of The Hypothesis, however, a plausible explanation is evident; namely, encounters between acarians and humans have increased.

Just as the prevalence of allergic diseases is increasing, so, too, is the prevalence of tick-borne illnesses, examples of which include galactose-α-1,3-galactose (α-gal) hypersensitivity and Lyme disease (5–12). Although the increasing prevalence of tick-borne illnesses has been attributed to climate change and deforestation (13, 14), these factors cannot account for the rise in IgE-mediated diseases because synanthropic mites thrive in the climate-controlled human habitats of the developed world, where allergies are especially prevalent.

According to The Hypothesis, the induction of an allergic disease requires that causative acarians be present on human epithelium (1–3). In keeping with α-gal sensitization, the number of infesting organisms need not be great (15–17). Given the phylogenetic relatedness of mites and ticks, it is reasonable to assume that a change to the epithelial environment would influence the prevalence of all acarian-induced diseases.

A review of primate species and the acarian parasites that infest them supports a role for the eccrine glandular system in the anti-acarian defense of humans. *Homo sapiens* are subject to permanent parasitism by two lineages of acarians, *Demodex* spp., which inhabit pilosebaceous units and subsist on sebum, and *Sarcoptes scabiei*, a carnivorous mite that burrows beneath skin (18, 19). Other primate species are subject to permanent parasitism by a third acarian lineage, *Psoroptidae*, a family of non-burrowing carnivorous mites (20).

Modern dwellings of *Homo sapiens* are infested by *Pyroglyphidae*, a family of polyphagous acarians whose diet includes, among other things, discarded epidermal materials (21, 22). The dust mites, *Dermatophagoides pteronyssinus* and *Dermatophagoides farinae*, belong to *Pyroglyphidae*. Materials expressed by them are intimately associated with allergic disease (23). Phylogenetic analysis suggests that *Pyroglyphidae* is a sister taxon to *Psoroptidae*, and that both families belong within Psoroptidia, a parvorder of mites, the representatives of which are almost exclusively parasitic (24). At some time in the past, *Pyroglyphidae* diverged from its sister taxon and followed an exceedingly unusual evolutionary pathway from parasite to free-living scavenger (24). Consequently, *Pyroglyphidae* consume fungi and bacteria, and they colonize and consume stores of human foodstuffs, e.g., wheat (25–28).

One possible explanation for the apparent resistance of *Homo sapiens* to direct parasitism by non-burrowing *Psoroptidae*—as well as the continued affinity of some *Pyroglyphidae* for discarded human epidermal materials—is that a change to the epidermal environment of humans forced *Pyroglyphidae* to evolve from parasite to scavenger. Inasmuch as the eccrine glandular system distinguishes the epidermis of humans from that of other primates, it may have forced adaptation of the mite. If so, anything that interferes with the operation/function of glandular secretions, e.g., hygiene, might permit reversion of *Pyroglyphidae* to their parasitic ancestral mode. Consistent with a role for *Pyroglyphidae* in IgE-mediated diseases, the skin of patients with atopic dermatitis (AD) hosts many more *Pyroglyphidae* than does the skin of healthy persons (17).

As will be argued, phylogenetic analysis of evolutionary adaptations of primate species suggests secretions of eccrine glands participate in anti-acarian immunity. Further, the habitual removal and/or functional disruption of these secretions by contemporary hygienic practices increase acarian—human interactions, thereby accounting for the concomitant rise in both modern-day acarian-induced tick-borne illnesses and IgE-mediated diseases.

## 2 Blood types, α-gal and eccrine glands

Outcomes related to blood transfusion are informative. Among human populations there exists variability in the expression of immunoreactive carbohydrate epitopes on proteins and lipids of the outer membrane of red blood cells (29). Whereas blood type A individuals express as immunodominant antigen, N-acetylgalactosamine, blood type B individuals express as immunodominant antigen, D-galactose. Because type A individuals express the A antigen, they do not generate anti-A antibodies. Consequently, they tolerate blood products bearing the A antigen, but not ones bearing the B antigen. Likewise, type B individuals tolerate blood products bearing the B antigen, but not ones bearing the A antigen (30). In short, self-expression of a potentially immunoreactive carbohydrate protects the individual from a deleterious immune response that would otherwise be triggered by infused materials bearing that immunoreactant.

In a mechanistic sense, a tick bite is akin to a blood transfusion, albeit on a smaller scale. Just as ticks transmit pathogens from a previous blood meal to a new host, so, too, do they transmit residual blood materials from previous hosts. And just as humans can express A and/or B antigens, other tick hosts express taxon-specific immunoreactive carbohydrates. Thus, any tick taking a blood meal from a non-human host has potential to expose a subsequent human host to foreign immunoreactants.

Importantly, except for catarrhine primates, all mammals express the immunoreactive carbohydrate, α-gal (31). α-Gal linkages are catalyzed by the enzyme, N-acetyllactosaminide α-1,3-galactosyltransferase, encoded by the gene *GGTA1* (32). Phylogenetic analysis indicates catarrhine primates underwent a loss-of-function mutation in *GGTA1* ∼20–30 million years ago. That loss resulted in an inability to catalyze α-gal linkages (33). It is believed elimination of this non-essential carbohydrate allowed the production of anti-α-gal antibodies, which, in turn, protected ancestral primates from a near-extinction event (34).

Assuming the mechanics of IgE-mediated immunity are conserved among all mammalian species and, as posited by The Hypothesis, α-gal hypersensitivity in susceptible hosts develops following tick-directed “infusion” of α-gal-bearing material (2), only catarrhine primates should develop α-gal hypersensitivity. This raises the possibilities: (1) evolutionary pressure from acarians drove the expression of α-gal in mammalian lineages, and (2) catarrhine primates that ceased to express α-gal evolved an alternative means by which to deal with acarians.

The phylogenetic record indicates that, following emergence of the loss-of-function mutation in *GGTA1*, catarrhine primates evolved a trait best appreciated in *Homo sapiens*: full body expansion of eccrine glands (35). As elaborated next, experimental evidence indicates eccrine gland secretions deter epithelial colonization by microorganisms. Observational evidence indicates this deterrence extends to acarians.

## 3 Sweat, dermcidin and anti-acarian immunity

In *Homo sapiens*, epidermal secretions derive from 4 glands: eccrine, apocrine, apoeccrine and sebaceous (36, 37). Given that all 4 glands secrete onto the same epithelial surface, they undoubtedly act in concert. Because only eccrine gland expansion accompanied the *GGTA1* loss-of-function mutation, what follows focuses solely on eccrine glands and their secretions.

Although many mammalian species have eccrine glands, the distribution of these glands in most mammals is limited to hand- and footpads, where they provide friction for mechanical gripping (38). In some species of platyrrhine primates, distribution of eccrine glands includes the tail, which is also used for gripping (35). In catarrhine primates, eccrine glands are distributed across the entirety of body surface area. Humans have 2–4 million eccrine glands, the most of any primate, with the greatest surface density being on palms and soles (36). Eccrine glands derive from embryonic ectoderm. Their number is fixed early in life (39). Consequently, the surface density of glands decreases with expansion of body surface area, e.g., normal growth and obesity (40, 41). Analyses of eccrine glands have focused primarily on thermoregulatory function, although recent focus includes the immunoregulatory functions of sweat content, especially antimicrobials, e.g., dermcidin (dcd) (42–44), and cytokines, e.g., IL-31 (45, 46). Interplay between IL-31 and keratinocytes is especially germane. Under normal physiological conditions, stratum corneum prevents IL-31 from stimulating keratinocytes (47). However, mechanical disruption of that barrier, as happens during acarian parasitism, exposes keratinocytes to the cytokine, stimulating the cells to recruit leukocytes characteristic of AD, e.g., eosinophils (48). In addition to eliciting inflammation, IL-31 stimulates itch (49). Thus, not only does IL-31 prompt localized inflammation at a site of barrier disruption, but it also facilitates mechanical removal of parasitic acarians by means of scratching. Indeed, in a mouse model of AD, the development of mite-dependent lesions occurs concurrent with IL-31-dependent pruritic inflammation (50, 51).

Eccrine glands include a secretory coil and a delivery duct (52). Hydrostatic pressure drives sweat generated in the secretory coil through the duct and onto the skin. The intraepidermal portion of the duct is termed the acrosyringium. The secretory coil is comprised of 3 cell types: myoepithelial, clear and dark (36). Myoepithelial cells maintain the structural integrity of the gland (53). Clear cells and dark cells generate sweat content. Clear cells contribute water, electrolytes and inorganic substances (54, 55). Dark cells, which contain Schiff-reactive granules, contribute glycoproteins and other macromolecules, including dcd, the most abundant protein in human sweat, Table 1 (56, 57).

**Table 1:** Major proteins of eccrine gland secretions (57).

Protein	% Total proteins
Dermcidin	46
Clusterin	17
Apolipoprotein D	15
Prolactin-inducible protein	8
Albumin	6

First isolated in 2001, dcd accounts for nearly half the protein in sweat of healthy individuals (57). Its precursor protein includes a 19-residue signal peptide, a 43-residue pro-domain and a 48-residue antimicrobial domain (43). Proteolytic processing of dcd within sweat generates a variety of peptides active against an array of pathogens (58–60).

Although the toxicity of dcd toward acarians has not been investigated, its toxicity toward bacteria has been. In sweat, dcd polymerizes, forming channels composed of dimeric trimers (61, 62). Such channels translocate within membranes of pathogenic bacteria, disrupting transmembrane potentials and causing bacterial death. Regarding acarians, dcd may operate similarly, disrupting the membranes of organelles critical to acarian survival, e.g., mitochondria. Alternatively, channels of dcd might compromise peritrophic membranes, rendering acarians vulnerable to naturally occurring toxins present in undigested foodstuffs (63–65).

Expressed primarily in sweat glands, dcd is also expressed in tears, breast milk and sebum (66–68). It is encoded by *DCD*, an orphan gene unique to primates (58). The phylogenetic record indicates species that evolved the loss-of-function mutation in *GGTA1* acquired *DCD* and underwent propagation of eccrine glands (31, 35). If the loss-of-function mutation in *GGTA1* made catarrhine primates vulnerable to α-gal hypersensitivity, then it seems likely acquisition of the *DCD* gene and expansion of the eccrine glandular system were direct responses to the evolutionary pressure exerted by that vulnerability.

Unlike other antimicrobial peptides, which are upregulated in response to inflammation and injury, dcd is expressed constitutively (69). This suggests it functions as a deterrent, preventing epidermal colonization by pathogenic microbes. Furthermore, dcd is remarkably stable, persisting on epidermis more than 72 h (70). Thus, epidermal surfaces should normally be covered by a substantial layer of microbial deterrent. Currently, however, humans living in developed countries consider sweat “unhygienic”: they wash routinely (generally using heated water and soap), their lives are mostly sedentary, and they live and work in air-conditioned environments. It stands to reason, that the layer of dcd on humans today is something significantly less than it was formerly, enabling a current situation that fosters epidermal colonization by deleterious organisms, including some acarians.

Because dcd is expressed by primates exclusively, investigation of *DCD* knockout phenotypes is lacking. However, there is one report that details the clinical manifestations of a human family with a hereditary loss-of-function mutation in *DCD* (71). As related there, affected persons are predisposed to hidradenitis suppurativa (HS), a dermatopathology of uncertain etiology. Importantly, HS is associated with elevated levels of IgE (72), the antibody class central to anti-acarian immunity. HS is also linked to demodicosis (73), further supporting a role for dcd in anti-acarian immunity.

## 4 Atopic dermatitis, acarians and sweat

AD is the most common chronic inflammatory disease of the skin. The incidence of AD is lowest in children born during the hot summer months, when sweating is common (74, 75). Epidermal surfaces of AD patients have a higher density of acarians than do those of healthy individuals (17, 76). *Demodex follicularis* and *Demodex brevis*, monoxenous acarians increased on the skin of AD patients, are absent from eccrine acrosyringia, suggesting even acarians adapted to human epidermis find the local environment of eccrine glands inhospitable (77).

AD impairs acetylcholine-mediated control of sweating by the sudomotor reflex (78), and prolonged latency of sympathetic skin responses in AD confirms dysfunctional sympathetic pathways in the disorder (79). Besides dysregulation of sweat volume, sweat content is also affected. Peptides derived from dcd are reduced in the sweat of AD patients (80). It is yet uncertain whether the impaired sudomotor reflex is etiological or whether it is a pathological consequence of disease. In either case, if, as proposed, dcd-containing sweat is cardinal to anti-acarian immunity, it is reasonable to assume that a decreased sudomotor reflex favors habitation of acarians on affected epidermal surfaces.

Multiple tick species have been shown to produce toxins that prevent pre-synaptic release of acetylcholine, a measure that inhibits muscle activation in mammals (81). Such inhibition paralyzes tick hosts precluding, at the very least, reflexive itching and self-removal of ticks. Should resident acarians similarly disrupt the cholinergic pathway controlling the sudomotor reflex, the resulting epidermal microenvironment would be one favorable to continued acarian colonization.

What follows next are brief descriptions of proposals by others that attempt to account for allergy on the basis of hygiene or epidermal barrier dysfunction. The observations that spawned those proposals are then re-interpreted in the context of The Acari Hypothesis. An argument is made that removal of sweat by current hygienic practices allows acarians, the operative agents of allergy, to flourish on epidermal surfaces and promote allergic disease.

## 5 Hygiene, allergy and the epidermal barrier in the context of the acari hypothesis

It was first recognized in 1989 that children of large families are less likely to develop hay fever and AD. Among the children of those families, the younger ones are less likely to develop the conditions (82). Initially, the reduced allergic burden was hypothesized to be due to an increased risk of infectious disease in childhood, a consequence of either: (1) transmission of pathogens from older siblings to younger ones, or (2) transmission of pathogens from mothers infected by their older children. As expressed more recently, modern hygiene reduces antigenic load thereby increasing susceptibility to allergic diseases (83). Unfortunately, studies since publication of the original proposal have not been supportive (84). Still, despite the perceived inadequacy of the hygiene proposal, the original observations that prompted its formulation remain valid and, until now, unexplained. In this regard, The Acari Hypothesis accounts for the observations that prompted the hygiene proposal; namely, hygienic practices that remove sweat increase epidermal infestation by certain acarians, which, in turn, increases the likelihood of allergic disease.

As noted earlier, the medical community did not appreciate the existence of allergic diseases until about 1870 (4). Around that same time, 1868, the first “water heater” was patented (85). Although acceptance and availability of water heaters by the general public were not immediate, the very existence of a patent for the device implies societal interest in bathing regularly in hot water.

The limited capacity of residential water heaters readily accounts for the association between sibship size and order, and allergic diseases. Given a finite supply of hot water, the volume available for bathing per family member goes down with increasing family size. Because the layer of sweat-derived materials on skin is inversely related to the availability of hot water for bathing, children of large families, especially the younger children, do not routinely “wash away” their replenishable anti-acarian barrier. Consequently, their skin is less accommodating to acarian habitation, lessening their risk of allergic disease.

The Acari Hypothesis also accounts for the many studies that demonstrate children raised on farms are at decreased risk of allergic diseases (86). Firstly, persons living in rural communities spend more time outside and use less air conditioning than their urban contemporaries (87, 88). Secondly, rural communities lack the resources necessary to support the level of hygiene practiced in urban settings (89). From these, it follows that children living in rural environments are both more likely to sweat and less likely to remove sweat than children living in urban environments. As a result, a more robust anti-acarian layer of sweat confers to children raised on farms resistance to the acarian interactions necessary for the development of allergic disease.

Finally, the mechanics of mite parasitism are consistent with another popular proposal on allergic disease, one that involves the barrier function of skin. Published in 2017, that proposal postulates that epidermal barrier dysfunction enables allergic sensitization and development of type 2 inflammatory disease (90). The proposal is supported by data that shows persons with mutations in epidermal structural proteins, most notably filaggrin, are at substantial risk for AD (91, 92). Filaggrin is a protein critical to the formation of corneocytes, the cells that comprise the outermost barrier of human epidermis (93). Mutations in filaggrin compromise the barrier function of the stratum corneum, thereby permitting greater penetration of antigenic material against which IgE is generated (94). In keeping with The Acari Hypothesis, pyroglyphid mites on human skin are causative agents of AD. Although the feeding habits of pyroglyphid mites on human epidermis have not been described, those of mites of their sister taxon, *Psoroptidae*, have been, and they are exceedingly informative (95). In order to feed, *Psoroptidae* first deposit antigenic material from their digestive tract onto the surface of their mammalian host. Subsequently, they abrade the host skin with their mouthparts, generating inflammation (95). The mites then feed on the resulting exudate. Importantly, secretions from the mite digestive tract contain the adjuvant-active immune complexes claimed essential per The Hypothesis (2). A weakened barrier, as occurs in persons with filaggrin mutation, undoubtedly allows for greater penetration of mite digestive secretions and, consequently, greater exposure to the adjuvant-active complexes that drive IgE formation.

## 6 Closing

The Acari Hypothesis posits that ancestral acarians exerted formative influence on evolution of *Homo sapiens* at least twice: once during the emergence of class Mammalia, the other during the emergence of catarrhine primates. Such influence likely prompted the evolution of accommodative adaptations in humans and other mammals, particularly adaptations involving epithelial surfaces. With specific regard to allergy, nonhuman mammalian species, e.g., cats, dogs, horses, etc., have evolved different adaptations to mitigate the acarian risk. Indeed, and as will be elaborated and discussed in the next installment of The Hypothesis, that risk brings clarity and uniformity to the nature of allergenicity (ACR, submitted). In the context of The Hypothesis, targeted experimentation should identify relevant human adaptations, the knowledge of which should, yield mechanistic understanding of many issues pertinent to human health and disease.

Despite sweat having already been recognized as an immunoregulatory fluid, its role in human immunity is still grossly underappreciated by scientists and clinicians alike. Although this report provides only anecdotal evidence of sweat as a deterrent to epidermal habitation by acarians, sweat is known to be active against a variety of microbes, including and especially *Staphylococcus aureus*. It appears disruption/removal of the microbial deterrent yields an “immunocompromised” state that enables epidermal colonization by many organisms, pathological and otherwise. If, indeed, this is the case, then it seems likely many disease processes, the emergences of which postdate contemporary hygienic practices, are consequences of disruption/removal of epidermal materials derived from eccrine glands. As one important candidate, the metabolic syndrome is eminently suited to such consideration and analysis (ACR, submitted).

## Data Availability

The original contributions presented in the study are included in the article/Supplementary Material, further inquiries can be directed to the corresponding author.
